# Analysis of Heavy Rainfall in Sub-Saharan Africa and HIV Transmission Risk, HIV Prevalence, and Sexually Transmitted Infections, 2005-2017

**DOI:** 10.1001/jamanetworkopen.2022.30282

**Published:** 2022-09-08

**Authors:** Jason M. Nagata, Karly Hampshire, Adrienne Epstein, Feng Lin, Jennifer Zakaras, Pamela Murnane, Edwin D. Charlebois, Alexander C. Tsai, Denis Nash, Sheri D. Weiser

**Affiliations:** 1 Department of Pediatrics, University of California, San Francisco; 2 Department of Medicine, University of California, San Francisco; 3 Department of Vector Biology, Liverpool School of Tropical Medicine, Liverpool, United Kingdom; 4 Department of Epidemiology and Biostatistics, University of California, San Francisco; 5 Center for Global Health and Mongan Institute, Massachusetts General Hospital, Boston; 6 Institute for Implementation Science in Population Health, City University of New York, New York

## Abstract

**Importance:**

Extreme precipitation, including heavy rains and flooding, is associated with poor health outcomes mediated in part by decreases in income and food production. However, the association between heavy rains and HIV burden is unknown.

**Objective:**

To investigate the association between heavy rainfall, HIV prevalence, and HIV transmission risk over a 12-year span in sub-Saharan Africa.

**Design, Setting, and Participants:**

A cross-sectional population-based study, using data collected from the 2005-2017 Demographic and Health Surveys, was conducted in 21 countries in sub-Saharan Africa and analyzed from July 29, 2021, to June 14, 2022.

**Exposures:**

Heavy rainfall was defined based on the extent to which annual rainfall deviated from the historical average (standardized precipitation index ≥1.5) at the enumeration area level.

**Main Outcomes and Measures:**

HIV, self-reported sexually transmitted infections (STIs), and number of sexual partners.

**Results:**

The study included 288 333 participants aged 15 to 59 years; 172 344 were women (59.8%), and 183 378 were married (63.6%). Mean (SD) age was 31.9 (10.0) years. Overall, 42.4% of participants were exposed to at least 1 year of heavy rainfall in the past 10 years. Each year of heavy rainfall was associated with 1.14 (95% CI, 1.11-1.18) times the odds of HIV infection and 1.11 (95% CI, 1.07-1.15) times the odds of an STI in the past 12 months. There was also an association between heavy rainfall and the reported number of sexual partners (incident rate ratio, 1.12; 95% CI, 1.10-1.15). The odds were greater for the association between heavy rainfall and HIV prevalence and STIs among participants aged older than 20 years and participants in rural areas.

**Conclusions and Relevance:**

The findings of this study suggest that heavy rainfall was associated with a higher HIV burden in sub-Saharan Africa. The association between heavy rainfall and STIs and number of sexual partners suggests that an increase in the risk of sexual transmission is a plausible mechanism for the observed findings around HIV prevalence. Heavy rainfall could also worsen food insecurity, increasing the risk of transactional sex, or cause damage to public health infrastructure, reducing access to STI education, HIV testing, and treatment.

## Key Points

**Question:**

Is there an association between heavy rainfall, HIV prevalence, sexually transmitted infections, and HIV transmission risk?

**Findings:**

In a cross-sectional analysis of national survey data from 21 countries in sub-Saharan Africa with historical rainfall data, including 288 333 participants aged 15 to 59 years, each year of heavy rains was associated with higher odds of HIV infection, sexually transmitted infections, and a higher number of sexual partners.

**Meaning:**

The findings of this study suggest that heavy rainfall is associated with higher HIV burden in sub-Saharan Africa.

## Teaser

This population-based cross-sectional study evaluates the association of heavy rainfall with prevalence of HIV and sexually transmitted infections and number of sexual partners in sub-Saharan Africa.

## Introduction

Climate change is a growing public health threat; an emerging body of literature reports associations with infectious and noncommunicable disease, mental health, malnutrition, and mortality.^1^ In the field of infectious disease, extreme weather events caused by climate change, such as flooding and drought, have been linked to a higher frequency of vector-, water-, and food-borne diseases and fungal infections.^1^ Furthermore, extreme weather events can cause food insecurity, human migration, and infrastructure disruption, all of which can be associated with poor health outcomes.^2^

There are few data on how extreme weather events may affect HIV acquisition and onward transmission. To date, more than 79 million people worldwide have been infected with HIV and nearly half of those infected have died from AIDS-related illnesses.^3^ In recent decades, sub-Saharan Africa has become the epicenter of the epidemic, accounting for 39% of all new infections.^4^ Both HIV and climate change disproportionately affect globally underdeveloped regions such as sub-Saharan Africa.^2^ Climate change may be linked to HIV acquisition and onward transmission risk through complex pathways, including greater migration, higher infectious disease prevalence, access to HIV testing and antiretroviral therapy, infrastructure erosion, and food insecurity.^2^ For example, food insecurity linked to extreme weather events may be associated with high-risk for sexual activity that puts people—disproportionately women—at higher risk of contracting HIV. Many studies from sub-Saharan Africa and other regions have reported an association between food insufficiency and inconsistent condom use with nonprimary partners, sex exchange, intergenerational sexual relationships, and a lack of control of sexual relationships.^5,6^ Furthermore, human migration from extreme weather events can enlarge sexual networks, amplifying the risk for HIV.^7^

One study on drought and HIV burden reported that, for every recent drought, HIV prevalence in HIV-endemic areas increases by 11%.^8^ Among young females in rural drought-affected areas of Lesotho, drought was associated with higher HIV prevalence, lower educational attainment, and higher risk for sexually transmitted diseases.^9^ However, to our knowledge, although these pathways have been explored for some extreme weather events, others, including heavy rainfall, have yet to be studied. Modeling studies project a large increase in flood frequency in some global regions, including eastern Africa, and floods have been associated with food insecurity, human migration, and disruption of infrastructure, all of which are associated with greater HIV risk.^2,10^

The objective of this study was to determine the association between heavy rainfall and HIV prevalence in 21 countries in sub-Saharan Africa from 2005 to 2017. Furthermore, we investigated associations between heavy rainfall and HIV transmission risk, including number of sexual partners and sexually transmitted infections (STIs). We hypothesized that heavy rainfall events are associated with increases in HIV prevalence, as well as higher risk of sexual transmission, with associations more prevalent among women and individuals living in rural areas.

## Methods

### Data Source and Participants

In a cross-sectional design, we combined survey data on 21 countries in sub-Saharan Africa participating in the Demographic and Health Surveys (DHS); the eTable in the Supplement provides country-specific surveys and years. Demographic and Health Surveys are cross-sectional, nationally representative household surveys conducted worldwide that use a stratified 2-stage cluster sampling design selecting enumeration areas and households within each enumeration area.^11^ All female individuals of reproductive age (15-49 years) from selected households are invited to participate in the survey. Male individuals aged 15 to 59 years are recruited from households included in the nationally representative household survey. Selection criteria are country-specific; in some surveys, all men are sampled and in others, a proportion of households (eg, a third to half of randomly selected among the nationally representative household sample) are selected for the men’s survey. The men’s age range is higher since men in the households of women of reproductive age (eg, partners) may have larger age ranges.

We included all eligible DHS from 2005 to 2017 (eTable in the Supplement) that included HIV testing and geolocated information on each enumeration area. We only included surveys that evaluated the outcomes and covariates of interest selected from nationally representative samples and included variation in heavy rainfall exposure in the study window. The study was conducted from July 29, 2021, to June 14, 2022. We followed the Strengthening the Reporting of Observational Studies in Epidemiology (STROBE) reporting guideline for cross-sectional studies. Because all data were obtained from published literature, this study does not constitute human participant research and does not require institutional review board review or exemption according to the US Department of Health and Human Services (45 CFR §46).

### Measures

#### Heavy Rainfall

Heavy rainfall was measured using the Standardized Precipitation Index (SPI) for each calendar year in each survey enumeration area. The SPI is a widely used tool for detecting and characterizing heavy rainfall anomalies and SPI data were accessed from the Columbia Climate School International Research Institute Climate Data Library.^12,13,14^ The SPI is the number of SDs that observed cumulative precipitation deviates from the climatologic average. The index is computed by fitting long-term raw precipitation time series data (since 1979) of 1 frequency distribution (eg, γ) to another probability distribution (eg, normal or gaussian), then calculating the number of SDs by which short-term precipitation data (past year) deviates from the long-term mean in a given area.^13,14,15^ Additional details about the SPI models and methods are described elsewhere.^13,14,16^ We generated a binary categorization of heavy rain, defined as an annual SPI greater than or equal to 1.5. Standardized Precipitation Index values above 1.5 correspond to moderately wet or extremely wet on the European Drought Observatory classification scheme.^13,17^ We estimated the count of years that each survey respondent was exposed to heavy rainfall in the 10 years before the survey date when the outcomes were measured. Heavy rainfall was estimated at the enumeration-area level. This process is similar to a study examining rainfall exposures and HIV outcomes,^8^ to maximize the chance that the exposure preceded the outcome.

#### HIV Prevalence and HIV Transmission Risk Behaviors

The prevalence of HIV was based on population-based laboratory HIV testing.^18^ The MEASURE DHS HIV testing protocol provides anonymous, informed, and voluntary testing of adults, which includes an initial enzyme-linked immunoassay (ELISA) test, and then retesting of all tests with positive results and 5% to 10% of those with negative results with a second ELISA. Among participants with discordant results on the ELISA tests, a new ELISA or Western Blot test is performed. All participants receive education materials and referrals for free counseling and testing. We considered additional outcomes selected before analysis, each representing a different dimension of HIV transmission risk.^19^ These outcomes included a binary indicator representing whether the respondent reported an STI in the 12 months before the survey date^20^ and a count of the number of sexual partners the respondent reported (other than the respondent’s spouse) over the previous 12 months. These DHS questions are consistent with standard indicators recommended for monitoring sexual activity in the context of HIV/AIDS programs.^21^ Similar self-reported sexual activity questions have demonstrated good validity and test-retest reliability.^22,23,24,25^

#### Covariates

We included several sociodemographic variables a priori that have theoretical and empirical associations with HIV acquisition and risk of transmission.^19,26^ These include sex (female vs male), marital status (married vs single, never married, widowed, or divorced), age (15-19, 20-29, 30-39, 40-49, ≥50 years), educational level (none, primary, secondary, and higher), household asset wealth,^27,28^ urban or rural residence, and a variable representing the survey calendar month, which accounted for seasonality. The wealth index is a measure of the household’s cumulative living standard and ownership of selected assets, which has been validated as a measure of economic status, performing better than the traditional consumption expenditure index.^27,28^

#### Subgroup Analyses

We assessed whether associations differed by population subgroups. These subgroups included sex, urban vs rural residence, and age group (adolescent [15-19 years], younger adult [20-29 years], and adult [≥30 years]).^29^

### Statistical Analysis

To assess the associations between heavy rainfall and HIV prevalence and risk of transmission, a multivariable regression model was fitted for each outcome and pooled across all countries and within each country. We fitted the pooled models with survey-level fixed effects and the heavy rainfall variable, as well as sex, marital status, age, educational level, wealth index, urban residence, and survey month. Fixed effects allow for the models to be run within country and accounts for key differences between countries and the timing of surveys. Standard errors account for clustering at the enumeration area level. For binary outcomes (HIV prevalence and STIs), a logistic distribution was assumed. Given that the binary outcomes were relatively uncommon (<10%), the odds ratio (OR) approximates the risk ratio.^30^ For the regression model specifying the number of sexual partners (count variable) as the outcome, we assumed a negative binomial distribution. The exponentiated coefficients from these models therefore represent the incidence rate ratio. To assess for interactions between heavy rainfall and potential modifiers (male vs female sex, rural vs urban residence, and adolescent vs younger adult vs adult age), we included product terms in the regression models and considered a 2-sided α significance level of .05 for the interaction term. Given that the evaluation of interactions was exploratory, we applied the Benjamini-Hochberg procedure to adjust for a false discovery rate.^31^ Subgroup analyses were conducted for ages 15 to 19 years given that HIV among this younger age group could be a proxy for HIV incidence because it captures a more recent pattern.^9^ All analyses were conducted in SAS, version 9.4 (SAS Institute Inc).

## Results

The analytic sample included 288 333 survey respondents from 21 countries. The sample comprised 172 344 women (59.8%) and 115 989 men (40.2%); mean (SD) age was 31.9 (10.0) years, more than half of the respondents (183 378 [63.6%]) were married, and more than two-thirds (194 065 [67.3%]) lived in rural areas. Table 1 reports additional sociodemographic characteristics and outcomes of respondents in the analytic sample. Overall, 122 154 participants (42.4%) were exposed to at least 1 year of heavy rainfall in the past 10 years. Few respondents were HIV-positive (20 300 [7.0%]) or reported STIs in the 12 months before the survey (13 318 [4.6%]). The mean (SD) number of sexual partners in the past 12 months (other than spouse) was 0.34 (1.25).

**Table 1.** Descriptive Statistics of 288 333 Survey Respondents Included in the Analysis

Variable	No. (%)
Sex	
Female	172 344 (59.8)
Male	115 989 (40.2)
Married	183 378 (63.6)
Age, y	
15-19	28 290 (9.8)
20-24	49 800 (17.3)
25-29	54 184 (18.8)
30-34	46 889 (16.3)
35-39	40 149 (13.9)
40-44	30 716 (10.7)
45-49	23 831 (8.3)
50-54	9030 (3.1)
55-59	5444 (1.9)
Education	
None	70 941 (24.6)
Primary	118 583 (41.1)
Secondary	85 164 (29.5)
Higher	13 645 (4.7)
Wealth quintile	
Poorest	57 478 (19.9)
Poorer	56 126 (19.5)
Middle	55 948 (19.4)
Richer	57 297 (19.9)
Richest	61 484 (21.3)
Rural residence	194 065 (67.3)
Exposure	
No. of years of heavy rainfall in past 10 y	
0	166 179 (57.6)
1	81 470 (28.3)
2	34 372 (11.9)
3	5729 (2.0)
4	583 (0.2)
Outcomes	
HIV prevalence	20 300 (7.0)
Sexually transmitted infections, past 12 mo	13 318 (4.6)
No. of sexual partners in the past 12 mo, mean (SD)	0.34 (1.25)
Median (IQR)	0 (0-1)

**Figure 1.**
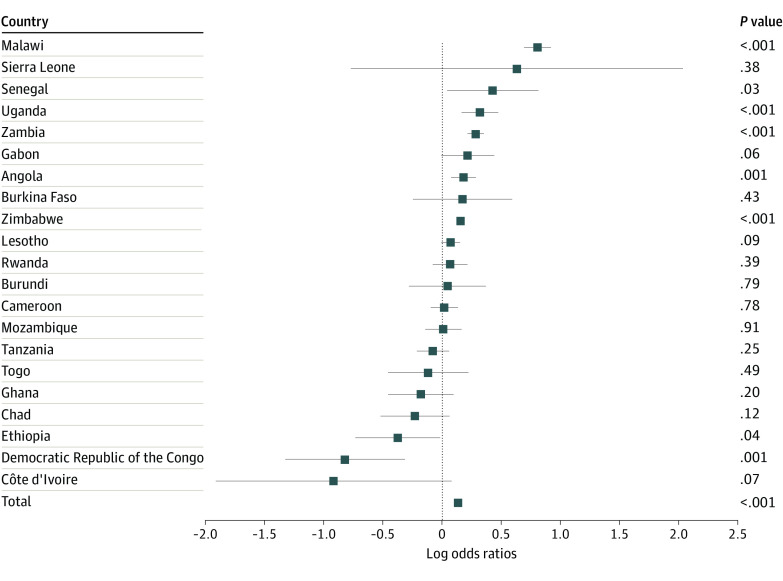
Country-Level Associations Among Heavy Rainfall and HIV Prevalence All models controlled for sex, marital status, age, educational level (none, primary, secondary, and higher), wealth index, urban residence, and survey month. Standard errors account for clustering at the enumeration area level.

**Figure 2.**
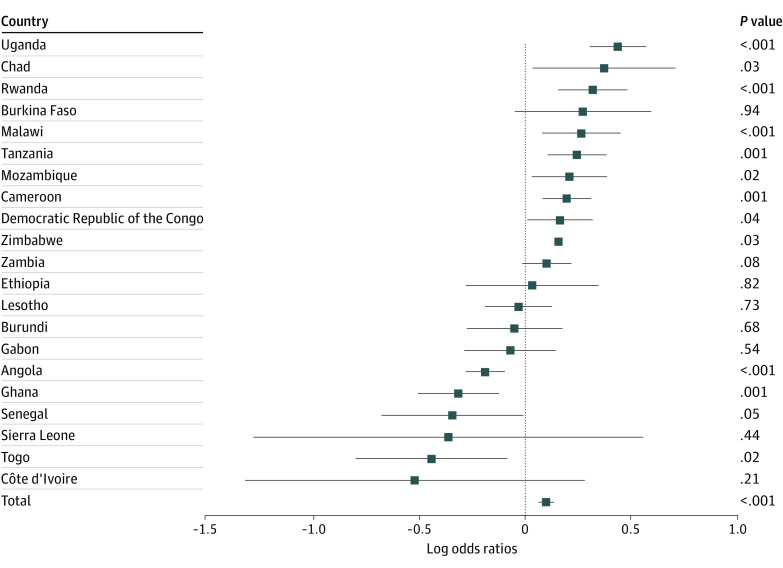
Country-Level Associations Among Heavy Rainfall and Sexually Transmitted Infections in the Past 12 Months All models controlled for sex, marital status, age, educational level (none, primary, secondary, and higher), wealth index, urban residence, and survey month. Standard errors account for clustering at the enumeration area level.

**Figure 3.**
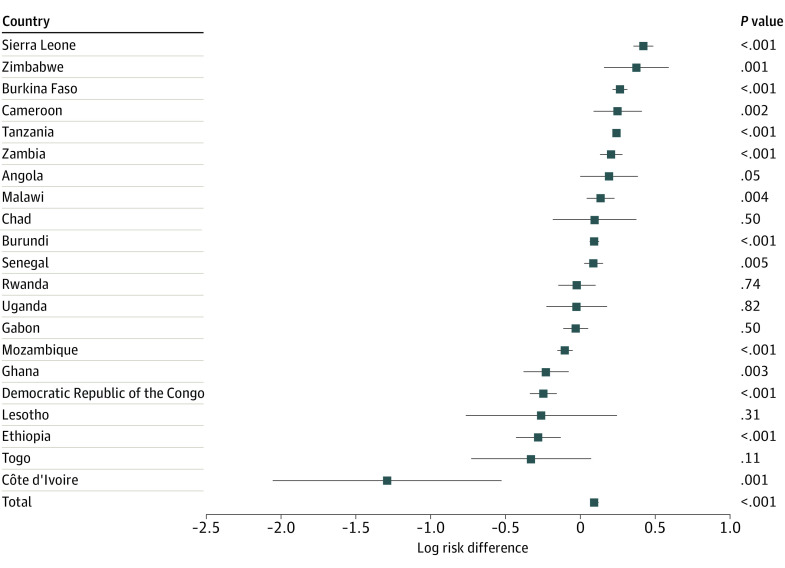
Country-Level Associations Among Heavy Rainfall and Number of Sexual Partners in the Past 12 Months All models controlled for sex, marital status, age, educational level (none, primary, secondary, and higher), wealth index, urban residence, and survey month. Standard errors account for clustering at the enumeration area level.

In logistic regression analyses (Table 2), each year of heavy rainfall was associated with 1.14 (95% CI, 1.11-1.18) higher odds of prevalent HIV infection and 1.11 (95% CI, 1.07-1.15) higher odds of STIs in the past 12 months. We found evidence of an association between heavy rainfall and the respondent’s reported number of sexual partners (incidence rate ratio, 1.12; 95% CI, 1.10-1.15). Figure 1, Figure 2, and Figure 3 show the estimates for the associations between heavy rainfall and the 3 outcomes of interest for each country individually. Subgroup analyses for participants aged 15 to 24 years are shown in eFigures 1-3 in the Supplement.

**Table 2.** Adjusted Estimates for the Associations Between Heavy Rainfall and HIV Prevalence and Sexual Risk Outcomes^a^

Variable	HIV prevalence		STI in the past 12 mo		No. of sexual partners in the past 12 mo	
	aOR (95% CI)	*P* value	aOR (95% CI)	*P* value	IRR (95% CI)	*P* value
Overall	1.14 (1.11-1.18)	<.001	1.11 (1.07-1.15)	<.001	1.12 (1.10-1.15)	<.001
Stratified by sex						
Male	1.12 (1.08-1.17)	<.001	1.09 (1.04-1.15)	<.001	1.11 (1.08-1.14)	<.001
Female	1.16 (1.12-1.20)	<.001	1.11 (1.07-1.16)	<.001	1.14 (1.10-1.18)	<.001
Interaction *P* value	.07		.43		.21	
Stratified by rural/urban						
Rural	1.25 (1.20-1.30)	<.001	1.14 (1.09-1.19)	<.001	1.12 (1.08-1.15)	<.001
Urban	1.02 (0.98-1.07)	.28	1.06 (1.01-1.11)	.01	1.13 (1.09-1.17)	<.001
Interaction *P* value	<.001		.008		.57	
Stratified by age category						
Adolescent (15-19 y)	1.00 (0.93-1.08)	>99	0.86 (0.78-0.94)	.002	1.04 (1.01-1.07)	.01
Younger adult (20-29 y)	1.11 (1.07-1.16)	<.001	1.07 (1.02-1.12)	.003	1.11 (1.08-1.14)	<.001
Adult (≥30 y)	1.17 (1.13-1.22)	<.001	1.18 (1.13-1.23)	<.001	1.21 (1.16-1.26)	<.001
Interaction *P* value	<.001		<.001		<.001	

Abbreviations: aOR, adjusted odds ratio; IRR, incidence rate ratio; STI, sexually transmitted infection.

^a^ All findings statistically significant after Benjamini-Hochberg procedure except HIV prevalence by urban residence and adolescent age. All models controlled for sex, marital status, age, educational level (none, primary, secondary, and higher), wealth index, urban residence, and survey month. Standard errors account for clustering at the enumeration area level.

Table 2 reports the adjusted estimates for the association between heavy rainfall and the 3 outcomes of interest (HIV, STIs, and number of sexual partners), stratified by sex, urban vs rural residence, and age group. We found evidence for multiplicative interaction. The adjusted ORs (aORs) for the association of heavy rainfall were greater for prevalent HIV in rural areas (aOR, 1.25; 95% CI, 1.20-1.30), in adults aged 30 years or older (aOR, 1.17; 95% CI, 1.13-1.22), and in younger adults (aOR, 1.11; 95% CI, 1.07-1.16). Similarly, the odds for the association of heavy rainfall with STIs were greater in rural areas (aOR, 1.14; 95% CI, 1.09-1.19), in adults aged 30 years or older (aOR, 1.18; 95% CI, 1.13-1.23), or younger adults (aOR, 1.07; 95% CI, 1.02-1.12). Heavy rainfall was associated with a higher number of sexual partners in adults aged 30 years or older (aOR, 1.21; 95% CI, 1.16-1.26) and in younger adults (aOR, 1.11; 95% CI, 1.08-1.14). We did not find evidence for effect modification of heavy rainfall by sex in the association with HIV prevalence, STIs, or number of sexual partners.

## Discussion

The findings of this study suggest that, among a large sample of people living in 21 countries in sub-Saharan Africa from 2005 to 2017, exposure to heavy rainfall was significantly associated with higher odds of HIV, with each additional year of experiencing heavy rainfall associated with 14% higher odds of having HIV. This association adds to the growing body of literature on the health outcomes of heavy rainfall, which include immediate events (eg, traumatic injury), midterm events (eg, infection and chemical contamination), and long-term events (eg, mental health disorders and malnutrition).^32^

In addition to being associated with HIV prevalence, heavy rainfall was associated with STIs and the number of sexual partners in the past 12 months, suggesting that an increase in the risk of sexual transmission could be at least partially responsible for the observed findings. Although the findings were significant, the relevance of many of the associations appears to be small to moderate. However, these associations may have public health and clinical relevance given that heavy rainfall is not uncommon and may become more frequent.

Heavy rainfall is known to deplete agricultural yields, and food insecurity is associated with an increase in sexual risk through transactional sex and the inability to broker safe sex.^32,33,34^ Therefore, one possible explanatory pathway for our findings is food insecurity leading to transactional sex and other economically motivated relationships as a means of paying for food, resulting in HIV infection.^35,36^ This theory is supported by the finding that the odds of increased HIV prevalence with heavy rainfall were greater in rural settings, where people are more likely to grow their own food and be directly reliant on crop yields. In addition, flooding and food insecurity may be associated with migration, leading to the expansion of sexual networks and creating conditions conducive to intravenous drug use or exposure to gender-based violence.^2^ Another possible mechanistic pathway for our findings is that flooding or excessive rains can block access to clinics or cause damage to public health infrastructure, weakening access to health resources such as pre-exposure prophylaxis and HIV testing, education, and treatment. This hypothesized pathway is speculative, however, given that there are no published findings to our knowledge.

Studies have reported that women are significantly more vulnerable to the health outcomes of extreme weather events.^9,37^ The association between heavy rainfall and HIV prevalence was pronounced in women (aOR, 1.16) compared with men (aOR, 1.12), although the *P* value for interaction was not statistically significant. Women have access to fewer income-earning opportunities than men and, thus, are more vulnerable to disruptions in income, exacerbating preexisting inequities.^38^ Investments that empower women and girls can also function as climate justice solutions by increasing reproductive autonomy and sustainable farming practices.^38^ However, it is useful for interventions to also target men, because men are less likely to access HIV prevention services compared with women despite reporting greater risk for sexual transmission. Because HIV transmission in sub-Saharan Africa generally occurs through heterosexual sex, HIV-prevention interventions that target men may also lower the prevalence among women.^39^

### Strengths and Limitations

This study has strengths and limitations. We leveraged large, population-based health data sets from 21 countries in sub-Saharan Africa that span more than a decade, as well as a high-resolution weather data set. One limitation is that because of a lower amount of temporal data, this study analyzed prevalent HIV infection rather than incident HIV infection. It is therefore challenging to discern whether heavy rainfall is associated with an increase in HIV acquisition, onward HIV transmission, or an increase in HIV survival. We created the heavy rainfall exposure to include the 10 years preceding each outcome specific to country and year to maximize the chance that the exposure preceded the outcome as has been defined in a previous analysis of climate exposures and HIV^8^; however, it is possible that participants could have acquired HIV more than 10 years earlier. It is also possible that there could have been a lag in HIV diagnosis such that heavy rainfall in the most recent year associated with HIV transmission may not yet have been diagnosed until the following period. Longitudinal research on heavy rainfall and HIV incidence is required to further characterize the association identified in our study.

Another limitation is that data on HIV transmission risk were self-reported in the DHS data set, which may be influenced by recall and social desirability biases, as well as the level of community stigma. Nonresponse bias may have affected results because HIV prevalence was based on population-based laboratory HIV testing. However, extensive analyses by studies that have used DHS data have not shown clear patterns in nonresponse rates by various risk or protective factors.^40^ Although our use of data spanning 12 years captures a greater number of participants and a wider range of weather variability, the large range of survey years may have led to variations in the observed effect estimates. The use of survey-level fixed effects may account for some of this variability and we do not hypothesize that the association between heavy rainfall and HIV prevalence would change over time. Although the measurements of rainfall are highly precise in the IRI/LDEO Climate Data Library,^12^ the distribution of satellite data and ground stations and the level of accuracy of the resultant data may not be consistent among countries, leading to the potential for misclassification of heavy rainfall in certain locations. However, by using the SPI, which categorizes heavy rainfall relative to the region-specific climatologic average, rather than an absolute threshold, we reduced the potential for misclassification. Residual confounding is a possibility, but deviations from long-term trends may be mostly independent of other potential confounding variables. In addition, our analysis only extends to 2017, excluding more recent HIV and rainfall data that may be influenced by progress in response to the UNAIDS 90-90-90 and 95-95-95 targets, and worsening climate change.^41^

## Conclusions

Given that HIV/AIDS is a leading cause of morbidity and mortality in sub-Saharan Africa and ongoing global warming is projected to amplify extreme weather events in the region,^42^ understanding associations between HIV/AIDS and extreme weather events is critical. Advancing research in this area may enable the tailoring of education and adaptation strategies.^43^ This study provides information that may be considered in designing HIV/AIDS prevention strategies, particularly for women, people living in rural settings, and adults older than 30 years in sub-Saharan Africa.

## Notes

Supplement. **eTable.** Country-Level Number of Years of Heavy Rainfall in Past 10 Years **eFigure 1.** Country-Level Associations Among Heavy Rainfall and HIV Prevalence, Ages 15-19 Years **eFigure 2.** Country-Level Associations Among Heavy Rainfall and Sexually Transmitted Infections in the Past 12 Months, Ages 15-19 Years **eFigure 3.** Country-Level Associations Among Heavy Rainfall and Number of Sexual Partners in the Past 12 Months, Ages 15-19 Years
