# Supplementary material for: Analysis of Heavy Rainfall in Sub-Saharan Africa and HIV Transmission Risk, HIV Prevalence, and Sexually Transmitted Infections, 2005-2017
Source: JAMA Netw Open. 2022 Sep 8;5(9):e2230282. doi: 10.1001/jamanetworkopen.2022.30282 (PMC9459663; doi:10.1001/jamanetworkopen.2022.30282)
Supplement: Supplement. — eTable. Country-Level Number of Years of Heavy Rainfall in Past 10 Years eFigure 1. Country-Level Associations Among Heavy Rainfall and HIV Prevalence, Ages 15-19 Years eFigure 2. Country-Level Associations Among Heavy Rainfall and Sexually Transmitted Infections in the Past 12 Months, Ages 15-19 Years eFigure 3. Country-Level Associations Among Heavy Rainfall and Number of Sexual Partners in the Past 12 Months, Ages 15-19 Years [file jamanetwopen-e2230282-s001.pdf]

Supplementary Online Content

Nagata JM, Hampshire K, Epstein A, et al. Analysis of heavy rainfall in sub-Saharan Africa and HIV transmission risk, HIV prevalence, and sexually transmitted infections, 2005-2017. *JAMA Netw Open*. 2022;5(9):e2230282. doi:10.1001/jamanetworkopen.2022.30282

eTable. Country-Level Number of Years of Heavy Rainfall in Past 10 Years

eFigure 1. Country-Level Associations Among Heavy Rainfall and HIV Prevalence, Ages 15-19 Years

eFigure 2. Country-Level Associations Among Heavy Rainfall and Sexually Transmitted Infections in the Past 12 Months, Ages 15-19 Years

eFigure 3. Country-Level Associations Among Heavy Rainfall and Number of Sexual Partners in the Past 12 Months, Ages 15-19 Years

This supplementary material has been provided by the authors to give readers additional information about their work.

© 2022 Nagata JM et al. *JAMA Network Open.*

eTable. Country-level number of years of heavy rainfall in past 10 years											
**Country**	**Survey years (DHS phase)**	**Number of years of heavy rainfall in past 10 years**									
		**0**		**1**		**2**		**3**		**4**	
		**n**	**%**	**n**	**%**	**n**	**%**	**n**	**%**	**n**	**%**
Angola	2015-2016 (7)	5785	62.5%	1449	15.7%	32	0.3%	1404	15.2%	583	6.3%
Burkina Faso	2010 (6)	7729	70.0%	3308	30.0%	0	0.0%	0	0.0%	0	0.0%
Burundi	2012 (6); 2016-2017 (7)	1626	11.2%	6692	46.1%	6007	41.4%	196	1.3%	0	0.0%
Cameroon	2011 (6)	5430	50.6%	536	5.0%	4762	44.4%	0	0.0%	0	0.0%
Chad	2014-2015 (7)	3911	50.5%	3012	38.9%	817	10.6%	0	0.0%	0	0.0%
Congo Democratic Republic	2013-2014 (6)	9809	70.9%	3465	25.0%	568	4.1%	0	0.0%	0	0.0%
Cote d'Ivoire	2011-2012 (6)	6900	98.2%	127	1.8%	0	0.0%	0	0.0%	0	0.0%
Ethiopia	2005 (5), 2016 (7)	13937	59.4%	8810	37.6%	708	3.0%	0	0.0%	0	0.0%
Gabon	2012 (6)	4900	53.2%	4319	46.8%	0	0.0%	0	0.0%	0	0.0%
Ghana	2016 (7)	4336	69.8%	855	13.8%	1018	16.4%	0	0.0%	0	0.0%
Lesotho	2009 (6), 2014 (7)	1247	12.8%	4185	43.0%	3419	35.1%	876	9.0%	0	0.0%
Malawi	2010 (6), 2015-2016 (7)	15529	73.6%	5567	26.4%	0	0.0%	0	0.0%	0	0.0%
Mozambique	2018 (7)	5768	63.7%	2726	30.1%	562	6.2%	0	0.0%	0	0.0%
Rwanda	2013 (6), 2014-2015 (7)	3533	22.9%	5729	37.2%	2976	19.3%	3183	20.6%	0	0.0%
Senegal	2010-2011 (6)	1038	16.9%	557	9.1%	4530	74.0%	0	0.0%	0	0.0%
Sierra Leone	2008 (5)	4555	99.0%	45	1.0%	0	0.0%	0	0.0%	0	0.0%
Tanzania	2007-2008 (5), 2010 (6)	17955	76.2%	4422	18.8%	1183	5.0%	0	0.0%	0	0.0%
Togo	2013-2014 (6)	2385	35.7%	4302	64.3%	0	0.0%	0	0.0%	0	0.0%
Uganda	2011 (6)	10446	68.4%	4831	31.6%	0	0.0%	0	0.0%	0	0.0%
Zambia	2007 (5), 2013-2014 (6)	22542	75.2%	5506	18.4%	1874	6.2%	70	0.2%	0	0.0%
Zimbabwe	2010-2011 (6), 2015 (7)	4269	20.1%	11027	52.0%	5916	27.9%	0	0.0%	0	0.0%

© 2022 Nagata JM et al. *JAMA Network Open.*

eFigure 1. Country-level associations among heavy rainfall and HIV prevalence, ages 15-19 years.

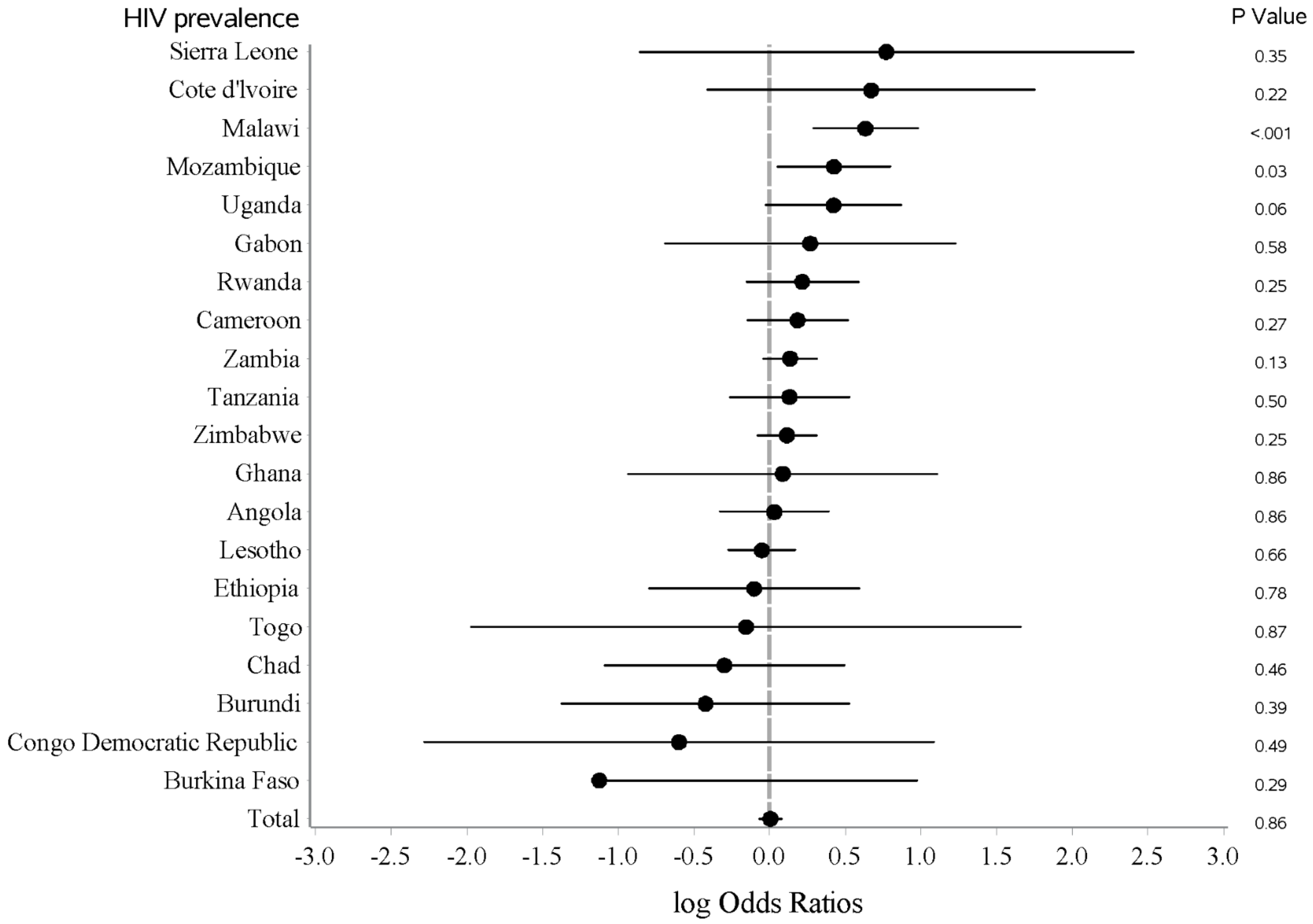

All models control for sex, marital status, age, education (none, primary, secondary, and higher), wealth index, urban residence, and survey month. Standard errors are clustered at the enumeration area level

© 2022 Nagata JM et al. *JAMA Network Open.*

eFigure 2. Country-level associations among heavy rainfall and sexually transmitted infections in the past 12 months, ages 15-19 years.

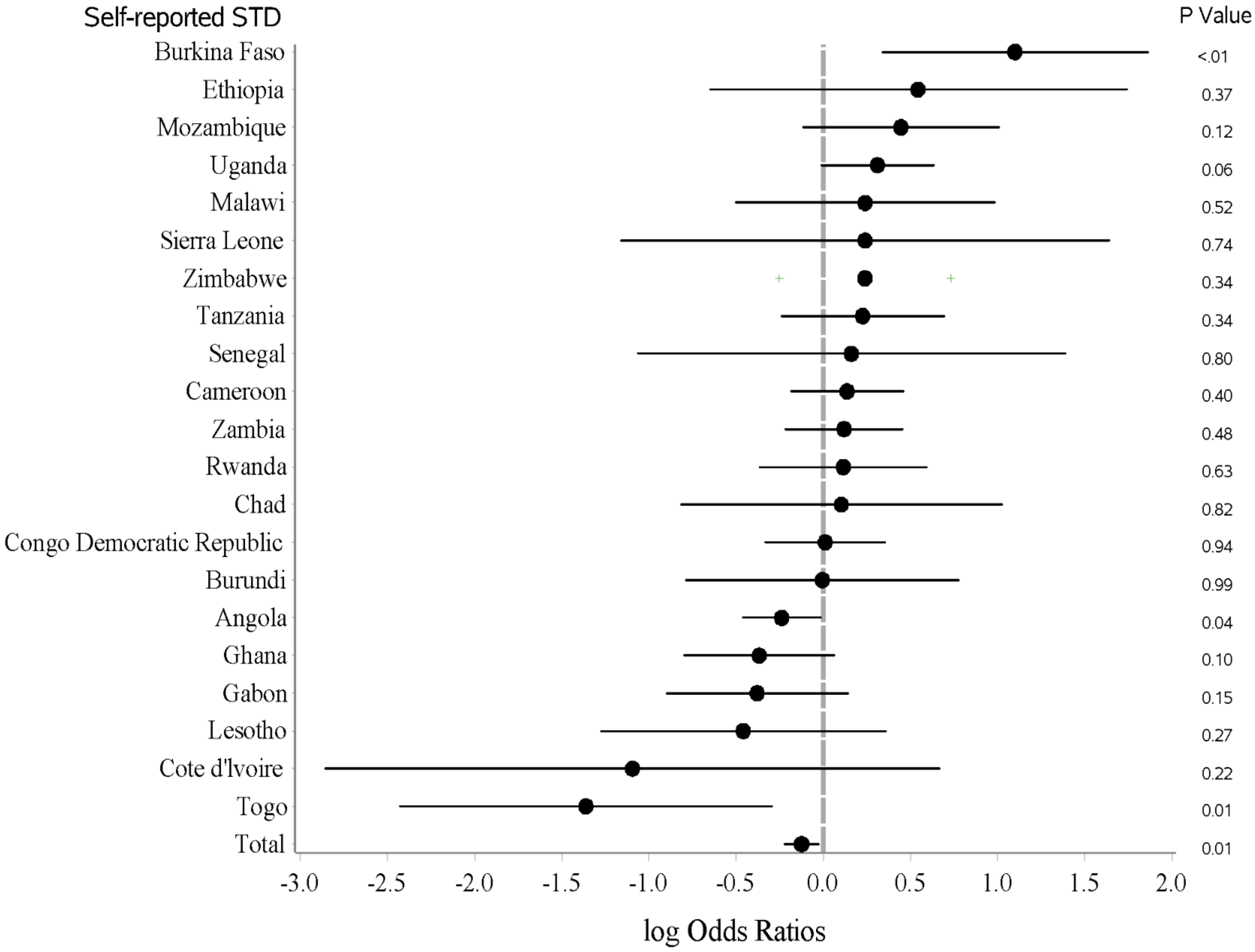

All models control for sex, marital status, age, education (none, primary, secondary, and higher), wealth index, urban residence, and survey month. Standard errors are clustered at the enumeration area level.

© 2022 Nagata JM et al. *JAMA Network Open.*

eFigure 3. Country-level associations among heavy rainfall and number of sexual partners in the past 12 months, ages 15-19 years.

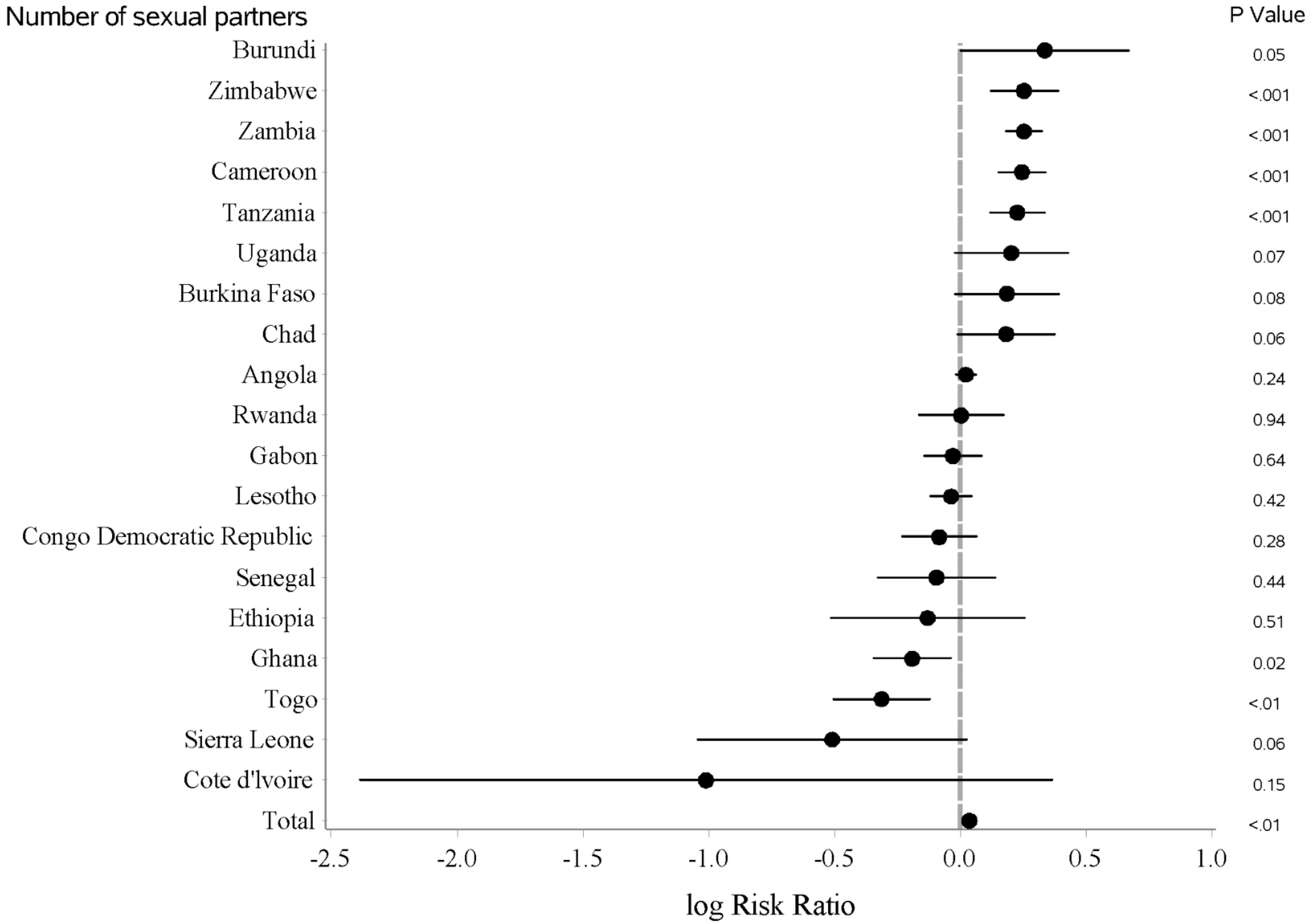

All models control for sex, marital status, age, education (none, primary, secondary, and higher), wealth index, urban residence, and survey month. Standard errors are clustered at the enumeration area level.

© 2022 Nagata JM et al. *JAMA Network Open.*
